# Trend of declining bed net utilization among pregnant women in Ethiopia: new data from the Arba Minch Health and Demographic Surveillance System, 2010–2016

**DOI:** 10.1186/s12936-020-03211-x

**Published:** 2020-04-08

**Authors:** Teklemariam Gultie, Gistane Ayele, Befikadu Tariku, Mekdes Kondale, Zerihun Zerdo, Behailu Merdekiyos, Tsegaye Tsalla, Mesfin Kote, Alemayehu Bekele, Mulugeta Shigaz, Gebrekiros Gebremichael, Feleke Gebremeskel, Alazar Baharu

**Affiliations:** 1 Department of Midwifery, College of Medicine and Health Sciences, Arba Minch University, Arba Minch, Ethiopia; 2 Department of Public Health, College of Medicine and Health Sciences, Arba Minch University, Arba Minch, Ethiopia; 3 Department of Medical Laboratory Science, College of Medicine and Health Sciences, Arba Minch University, Arba Minch, Ethiopia; 4 Ethiopian Public Health Association, Addis Ababa, Ethiopia; 5 Department of Computer Sciences, Arba Minch University, Arba Minch, Ethiopia

**Keywords:** Bed net, Malaria, Pregnant, Factors, Utilization

## Abstract

**Background:**

Bed net utilization is one of the important methods of malaria prevention. Malaria during pregnancy is one of the fatal diseases which mostly leads to the death of the mother and the fetus. Some of the complications of malaria during pregnancy are: intrauterine growth restrictions, intrauterine fetal death, and stillbirth. The main challenge of malaria treatment is that most of the anti-malarial drugs are not safe to use during pregnancy. The use of bed net is the most effective method of prevention of malaria during pregnancy. There is a paucity of information on bed net utilization among pregnant women in the study setting. Hence, this study aims to assess the trends of bed net utilization among pregnant women in Arba Minch Health and Demography Surveillance Site (HDSS), Southern Ethiopia.

**Methods:**

The study was conducted in the Arba Minch HDSS. The observation started in 2010 till 2016, using a repeated cross-sectional study design. The data was collected using interviewer administered questionnaire biannually with a total of 14 rounds of data collection from 2010 to 2016. A total of 2657 pregnant women were included in the study. Descriptive statistics such as frequency and proportion were used to present the findings of each variable.

**Results:**

Out of 2657 mothers included in the study, more than half, 1521 (63.6%), of the study participants were in the age group between 20 and 29 years. About one-third of the study population 793 (29.8) were having no schooling. The trend of bed net utilization decreased from 83.6% in 2010 to 36.5% in 2016.

**Conclusion:**

The trends of bed net utilization decreased from 2010 to 2016 in Arba Minch HDSS. Utilization of bed net by pregnant women in the area need to be increased as it is malaria endemic. The government should strengthen the existing bed net distribution strategy. Further research is needed to investigate the cause of decreasing bed net utilization.

## Background

Malaria is the leading cause of morbidity and mortality in many developing countries. In 2018, an estimated 228 million cases of malaria occurred worldwide. An estimated 405,000 deaths due to malaria occurred in 2018 due to malaria with more than 94% of the malaria deaths worldwide occurring in African region [1]. Each year, approximately 50 million women living in malaria-endemic countries throughout the world become pregnant estimated 10,000 of these women and 200,000 of their infants die as a result of malaria during pregnancy [2]. Malaria remains the leading public health problem in Ethiopia. About 75% of the landmass of the country is malaria risk areas and 68% of the population are living in such settings [3].

Bed nets are one of the proven cost-effective components of malaria prevention through vector control approach. The use of a bed net during pregnancy is shown to reduce miscarriages and stillbirths by 33% [4]. Bed nets have been known to reduce numbers of infective mosquito bites by 70 to 90% in various geographical settings [5]. In Africa, bed nets compared with no nets reduced placental malaria in all pregnancies, reduced low birth weight and fetal loss in the first to fourth pregnancy [6]. Studies in Western Kenya had shown that bed nets were associated with reductions in the incidence of malaria parasitaemia and incidence of severe malarial anaemia [6]. Bed nets have been shown to be the most cost-effective measures in the prevention of malaria [2].

The prevention and control of malaria during pregnancy is, therefore, crucial as it helps to promote the health of the mother and her unborn child. Utilization of bed net among pregnant women varies from 15.8% in Shashogo District, Southern Ethiopia to 90.5% in Enugu, South Eastern Nigeria [8, 9]. Studies in different areas of Nigeria showed that utilization of bed nets among pregnant women is 44.2% in Ibadan, 35.3% in Imo, 21.3% in Edo state, and 44% nationwide [9–12]. Utilization of bed nets among pregnant women in Bungoma County Kenya is 82.5%, Kilifi district, Kenya is 70.5%, in Gulu Uganda is 35%, in the Democratic Republic of the Congo is 78.4%, in the Buea Health District, Cameroon is 83.4%, Ghana is 20%, and Sudan is 11.5% [12–18]. In different parts of Ethiopia bed net utilization among pregnant women indicated that 72.5% in Damot Pulasa District, 23.2% in Oromia and Amhara Region, 73.3% in Eastern Ethiopia, 52.3% in Itang, Gambella region [3, 7, 9, 19]. The HDSS site is one of the malaria endemic area in the southern region of Ethiopia in which pregnant women and children are affected. The finding of the study will be used as an input for the government to further strengthen the already existing strategies of malaria prevention as well as a means for monitoring and evaluation of the performance of the government on bed net distribution. Therefore, this study aimed at assessing the trends of bed net utilization among pregnant women in Arba Minch HDSS from 2010 to 2016.

## Methods

### Study setting and period

The study was conducted in the Arba Minch Health and Demographic Surveillance System (HDSS) which was established in collaboration between Arba Minch University and Ethiopian Public Health Association with the support of CDC Ethiopia in 2009 with the aim of tracking demographic changes like death, birth, migration, and marital status change. The surveillance operates in the nine of 29 *kebeles* (smallest administrative unit) of Arba Minch Zuria District which is located in Gamo Zone, Southern Ethiopia. Arba Minch, the administrative town of the district is 505 km away from Addis Ababa in the southwest direction. Eight of the kebeles were selected based on the altitude (4 lowlands, 3 midlands and 1 highlands) and one of the kebele was selected as semi-urban. The total population of the Arba Minch HDSS in 2009 and in 2015 was 65,057 (49.41% female) and 71,890 (49.81% female), respectively. The data were collected from January 2010 to December 2016.

### Study design and sample size

A community based repeated descriptive cross-sectional study was conducted in the Arba Minch HDSS. The Arba Minch HDSS is a set of operations that longitudinally follow well-defined entities or primary subjects (individuals, households, and residential units) and all related demographic and health-related outcomes within a clearly defined geographic area. The Arba Minch HDSS site follows-up every individual within a defined catchments area two times a year with house-to-house visits. All pregnant women living at Arba Minch HDSS were followed from 2010 to 2016. During this period, a total of 2657 pregnant mothers included in the study. The number of pregnant women included in each year are 214 in 2010, 340 in 2011, 439 in 2012, 368 in 2013, 212 in 2014, 460 in 2015, and 624 in 2016.

### Data collection procedure

The data was collected using a structured interviewer administered questionnaire. The questionnaire was prepared by reviewing relevant literature and translated into the local language. The questionnaire included different socio-demographic and maternal health related variables as well as questions on the presence of bed net, the use of bed net and how they are using. Individuals and households have given identification numbers during the baseline enumeration and events updates are conducted biannually.

### Data quality assurance

Data collectors and supervisors were trained on the data collection process and tools. The data collection process has been closely supervised by the field supervisors and the research team. Field workers (data collectors and supervisors) were from the local community members who were fluent in the local languages and who had completed at least secondary school education. Data collectors checked their own completed forms before they handed them over to their supervisors. The field supervisors checked the filled questionnaires and provided feedback to the data collectors. Moreover, data entry was made by data entry clerks with close supervision of data managers.

### Statistical analysis

The data entered into the household registration system (HRS) 2 database system by data clerks and exported to Statistical package for the social sciences (SPSS) window version 25.0 statistical software. Final data cleaning was done by computing frequency and exploration through a whisker box plot for outliers. Descriptive statistics computed to determine the trend of bed net utilization. The result presented with text, tables, and graphs.

## Results

### Socio-demographic characteristics of the study participants

In this study, 2657 pregnant women were included. The mean age of the study population was 26 ± 5 years. The majority (63.6%) of the pregnant mothers were in the age group of 20–29 years old. One-third of the mothers (29.8%) have no schooling. One thousand six hundred seventy-six (63.1%) of the mothers were housewives. Out of the 2657 pregnant women, 2439 (91.8%) of the mothers were married. One thousand nine hundred fifteen (72.1%) pregnant mothers were rural residents (Table 1).

**Table 1:** Sociodemographic characteristics of pregnant women in Arba Minch HDSS, 2018 (N = 2657)

Variables	Response categories	Frequency	Percent
Age in years	15–19	249	10.4
	20–29	1521	63.6
	30–39	571	23.9
	> 40	49	2.1
Ethnicity	Gamo	1855	69.8
	Zeyise	193	7.3
	Wolayita	235	8.8
	Other^a^	374	14.0
Educational status	No schooling	793	29.8
	Primary	1691	63.7
	Secondary or above	173	6.5
Marital status	Married	2439	91.8
	Single	218	8.2
Religion	Protestant	2061	77.6
	Orthodox	558	21.0
	Other^b^	38	1.5
Residence	Rural	1915	72.1
	Urban	742	27.9

^a^Amhara, Oromo, Gofa

^b^Catholic, Muslims, atheists

### Trends of bed net utilization

**Fig. 1:**
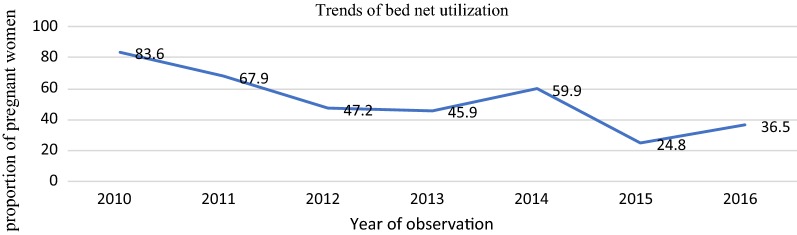
Trend of bed net utilization among pregnant women in Arba Minch Zuria HDSS from 2010 to 2016

On average from 2010 to 2016 only 1255 (47.2%) pregnant women utilized a bed net. The use of bed net over time starting from 2010 to 2016 decreased from 83.6 to 36.5% (Fig. 1).

## Discussion

The aim of this study was to assess the trends of bed net utilization among pregnant women in Arba Minch. The 7 years of data (2010–2016) were analysed. One thousand two hundred fifty-five (47.2%) pregnant women utilize the bed net. This finding is in line with the study finding in Northern Uganda (35%) [11]. However, the finding is lower than the study finding from Bungoma Kenya (82.5%), South Eastern Nigeria (90.5%), Kilifi Kenya (70.5%), Democratic Republic of Congo (78.4%), in Eastern Ethiopia (62.4%), in Itang Ethiopia (52.3%) [9, 10, 13, 14, 16, 20, 21]. The possible explanation for the discrepancy could be the government of Ethiopia has invested a lot to eradicate malaria, as a result, the magnitude of malaria infection in Ethiopia declined which make them give less attention to malaria prevention and the use of bed nets.

The overall trends of bed net utilization from 2010 to 2016 decreased from 83.6 to 36.5%. However, after 2015, there was increasing utilization of bed nets among pregnant women. The reason for increasing bed net utilization could be due to a malaria epidemic in the study area in 2015.

## Conclusion

The trend of bed net utilization among pregnant women decreased between 2010 to 2016. Pregnant women in the area need more supply and awareness of bed net utilization. The federal ministry of health should strengthen the existing bed net distribution strategies and monitor the utilization of bed net by the community. Further research is needed to investigate the cause of decreasing bed net utilisation.

## Data Availability

The datasets used and/or analysed during the current study are available from the corresponding author on reasonable request.

## Abbreviations

HDSS: Health and Demographic Surveillance System
HRS: Household registration system
SPSS: Statistical package for social sciences
